# Targeting human epidermal growth factor receptor 2 enhances radiosensitivity and reduces the metastatic potential of Lewis lung carcinoma cells

**DOI:** 10.1186/s13014-020-01493-8

**Published:** 2020-03-06

**Authors:** Yun Tien, Chiao-Ling Tsai, Wei-Hsien Hou, Yun Chiang, Feng-Ming Hsu, Yu-Chieh Tsai, Jason Chia-Hsien Cheng

**Affiliations:** 1 Division of Radiation Oncology, National Taiwan University Hospital, No. 7, Chung-Shan South Rd, Taipei, Taiwan; 2 Department of Psychiatry, Taoyuan Psychiatric Center, Taoyuan city, Taiwan; 3 Graduate Institutes of Clinical Medicine, National Taiwan University College of Medicine, Taipei, Taiwan; 4 Department of Radiation Oncology, City of Hope Medical Center, Duarte, CA USA; 5 Graduate Institutes of Oncology, National Taiwan University College of Medicine, Taipei, Taiwan; 6 Department of Oncology, National Taiwan University Hospital, Taipei, Taiwan

**Keywords:** Human epidermal growth factor receptor 2, Lung cancer, Radiotherapy, Afatinib, MMP-9

## Abstract

**Background:**

Sublethal radiation induces matrix metalloproteinase 9 (MMP-9)-mediated radioresistance in Lewis lung carcinoma (LLC) cells and their metastatic dissemination. We aim to determine if EGFR/HER2 activation associates with MMP-9-mediated radioresistance and invasiveness in irradiated LLC cells.

**Methods:**

LLC cells were treated with erlotinib or afatinib followed by sublethal radiation. After irradiation, we examined the phosphorylation of EGFR/HER2 and MMP-9 expression. Colony formation assay determined if the kinase inhibitors sensitize LLC cells to radiation. Matrigel-coated Boyden chamber assay assessed cellular invasiveness. Resulting tumors of wild-type LLC cells or HER2 knock-down mutant cells were irradiated to induce pulmonary metastases.

**Results:**

Afatinib more effectively sensitized LLC cells to radiation and decreased invasiveness by inhibiting phosphorylation of EGFR, HER2, Akt, ERK, and p38, and down-regulating MMP-9 when compared to erlotinib. Afatinib abolished radiation-induced lung metastases in vivo. Furthermore, LLC HER2 knock-down cells treated with radiation had growth inhibition.

**Conclusion:**

Dual inhibition of radiation-activated EGFR and HER2 signaling by afatinib suppressed the proliferation and invasion of irradiated LLC cells. Increased radiosensitivity and decreased metastatic dissemination were observed by pharmacological or genetic HER2 inhibition in vivo. These findings indicate that HER2 plays a pivotal role in enhancing radioresistance and reducing metastatic potential of LLC cells.

## Background

Radiation therapy (RT) improves the prognosis of locally advanced lung cancer. However, most patients succumb to distant metastasis [1]. Previous studies showed that a sublethal RT dose induces the up-regulation of matrix metalloproteinase 9 (MMP-9), which promotes cancer cell survival and metastasis [2]. The upstream targets that promote the up-regulation of MMP-9 in irradiated lung cancer cells remain unclear.

The epidermal growth factor receptor (EGFR) family is a group of transmembrane proteins that affect tumor cell viability [3]. As proto-oncogenes, overexpressions of EGFR families are found in multiple cancers. These genes play important roles in tumor progression by helping cells escape apoptosis and by promoting DNA repair and malignant cell metastasis [4]. Radiation stimulates the dimerization and auto-phosphorylation of EGFR family proteins and activates downstream signaling pathways [5–8]. Prior clinical investigations showed that EGFR inhibition sensitizes cancer cells to RT and improves locoregional cancer control [9].

For EGFR mutated NSCLC, EGFR tyrosine kinase inhibitors (TKIs) including afatinib and erlotinib show higher response rates and longer progression-free survival than platinum-based chemotherapy [10]. Afatinib covalently binds to EGFR, HER2, and HER4, and irreversibly inhibits tyrosine kinase autophosphorylation and downregulates ErbB signaling. Compared with erlotinib, a reversible EGFR tyrosine kinase inhibitor, afatinib has a broader spectrum with a theoretically better radio-sensitizing effect on cancer cell survival and a lower risk of metastasis [11]. Although the clinical efficacy of TKIs is well-established in patients with NSCLC, the benefit of combining EGFR TKIs with RT in this population remains uncertain.

We hypothesize that sublethal radiation activates EGFR and HER2, which subsequently up-regulates MMP-9 and associates with lung cancer cell survival and invasiveness. In this study, we showed that sublethal radiation doses increase phosphorylation of EGFR, HER2, and downstream Akt, ERK, and p38, and increase MMP-9 production in Lewis lung carcinoma (LLC) cells in vitro. Dual inhibition of radiation-induced EGFR and HER2 activation with afatinib strongly inhibited MMP-9 up-regulation and cell invasiveness in vitro and abolished pulmonary metastases in vivo in mice. HER2 inhibition with afatinib or its knock-down sensitized cancer cells to sublethal radiation.

## Methods

### Cell lines and cultures

The murine LLC cell line was obtained from the American Type Culture Collection. Cells were cultured at 37 °C in a humidified atmosphere of 5% CO_2_ and 95% air. Cell cultures were maintained in DMEM supplemented with 10% fetal bovine serum and penicillin/ streptomycin.

### Radiation treatment

LLC cells cultured in flasks were irradiated with different doses of radiation (0-10Gy), using a Cobalt-60 unit. The distance from the radiation source to the bottom of the flask was set at 80 cm. The dose rate was around 1 Gy/minute.

### Reagents

Afatinib and erlotinib were both purchased from Selleck Chemicals (Houston, TX). Afatinib and erlotinib were prepared in DMSO and 50% acetonitrile, and further diluted in culture medium before dosing for in vitro experiments. Both of them were suspended in a vehicle [0.5% methylcellulose (w/v) and 0.4% Tween 80 (v/v) in sterile water] for oral administration. For in vivo experiments, a daily dose of 10 mg/kg for afatinib or 50 mg/kg for erlotinib was administered to C57BL/6 mice (*n* = 42) bearing tumors for 7 days.

### Western blot analysis

Aliquots of cell lysates containing the protein extracts were loaded in each lane and separated by SDS-PAGE (8–15% polyacrylamide). After blocking, the membranes were probed with various antibodies. Bound antibodies were detected using the appropriate peroxidase-coupled secondary antibodies followed by the enhanced chemiluminescence detection system.

### Gelatin zymography

The supernatant of LLC cells (5 μl) was analyzed by sodium dodecyl sulfate–polyacrylamide gel electrophoresis on 10% polyacrylamide gels containing 1 mg/ml gelatin. The detailed method was previously described [12].

### Reverse transcription-polymerase chain reaction

The detailed method was previously described [12]. Specific gene cDNA was cloned and amplified by PCR with following primers: β-actin (sense 5′-CTCCTATGTGGGTGACGAGG-3′ and antisense 5′-CTTTTCACGGTTGGCCTT-3′ amplified a 202-bp fragment), and mouse MMP-9 (sense 5′-AACCCTGTGTGTTCCCGTT-3′ and antisense 5′-GGATGCCGTCTATGTCGTCT-3′ amplified a 486-bp fragment).

### Boyden chamber invasion assay

A total of 10^5^ cells were added to the upper chamber of invasion chamber inserted 50 μl (10 mg/ml) of Matrigel (Becton-Dickinson, Bedford, MA). After cell attachment, the medium was changed to serum-free medium, with each drug added for 30 mins, and the cells were irradiated. The detailed methods were previously described [12]. The experiments were repeated for three times.

### Colony formation assay

LLC cells (1 × 10^3^/well) were cultured in 6-well plates, treated with different doses of radiation following a 1-h pretreatment with afatinib or erlotinib on day 1, incubated for 7 days, and stained with 0.5% crystal violet in 10% methanol for 30 min at room temperature. The number of colonies (clusters of more than 50 cells) was counted in each well using an inverted phase-contrast microscope at 100x magnification and photographed. The experiments were repeated for three times.

### HER2 RNAi and stable transfection

To knock down HER2 gene expression, we used a target-specific lentiviral vector plasmid encoding a 19–25 nt hairpin shRNA (Santa Cruz Biotechnology; cat. no. sc-29,405-SH). The methods were previously described [12]. The efficiency of the HER2 knockdown (HER2-KD) in LLC cells was confirmed by Western blot analysis.

### In vivo ectopic tumor model

Male, 5- to 6-week-old, C57BL/6 mice (National Taiwan University Animal Center, Taipei, Taiwan) were used. Ectopic tumors were established by subcutaneous injection of LLC cells (1 × 10^6^) into right hind limb of mice. At 8 days after implantation, mice were immobilized in a customized harness that the right hind leg exposed. The thigh tumor was irradiated with five 10-Gy fractions on days 8–12 with a linear accelerator (Elekta Oncology System Ltd., Crawley, West Sussex, UK). Small animal positron emission tomography (PET)/ computed tomography (CT) scans with [^18^F]-2-fluoro-2-deoxyD-glucose (FDG) were performed on days 9 and 11. All the animal care, handling procedures, and experimental protocols were approved by the Committee of Experimental Animal Management at College of Medicine, National Taiwan University. The detailed methods were previously described [13].

### Histological evaluation and interpretation

Mice from each group were sacrificed on day 10. The tumor was fixed in 10% neutral buffered formalin and processed for histopathological and IHC staining. After fixation, tumor tissues were embedded in paraffin blocks and sectioned (10 μm). Tumor cells were identified in representative stained sections. The expressions of HER2 (Roche, Ventana, PA) and MMP-9 (BioSB, Santa Barbara, CA) were evaluated after immunohistochemical staining using specific antibodies. All images were digitally captured on an AxioImager. M1 (Zeiss) under 100x field and imaged. The color deconvolution tool, a plugin of TMARKER software [14], was used to count the total cell counts under the fields and the indicated stained cells.

### Statistical analysis

Data were presented as the mean ± standard deviation for the indicated number of separate experiments. Differences between pairs of treatment group were tested using the Student’s *t*-test, and a *p* value less than 0.05 was considered statistically significant.

## Results

### Inhibition of EGFR and HER2 tyrosine kinases inhibits radiation-activated MMP-9 transcription and translation

**Fig. 1:**
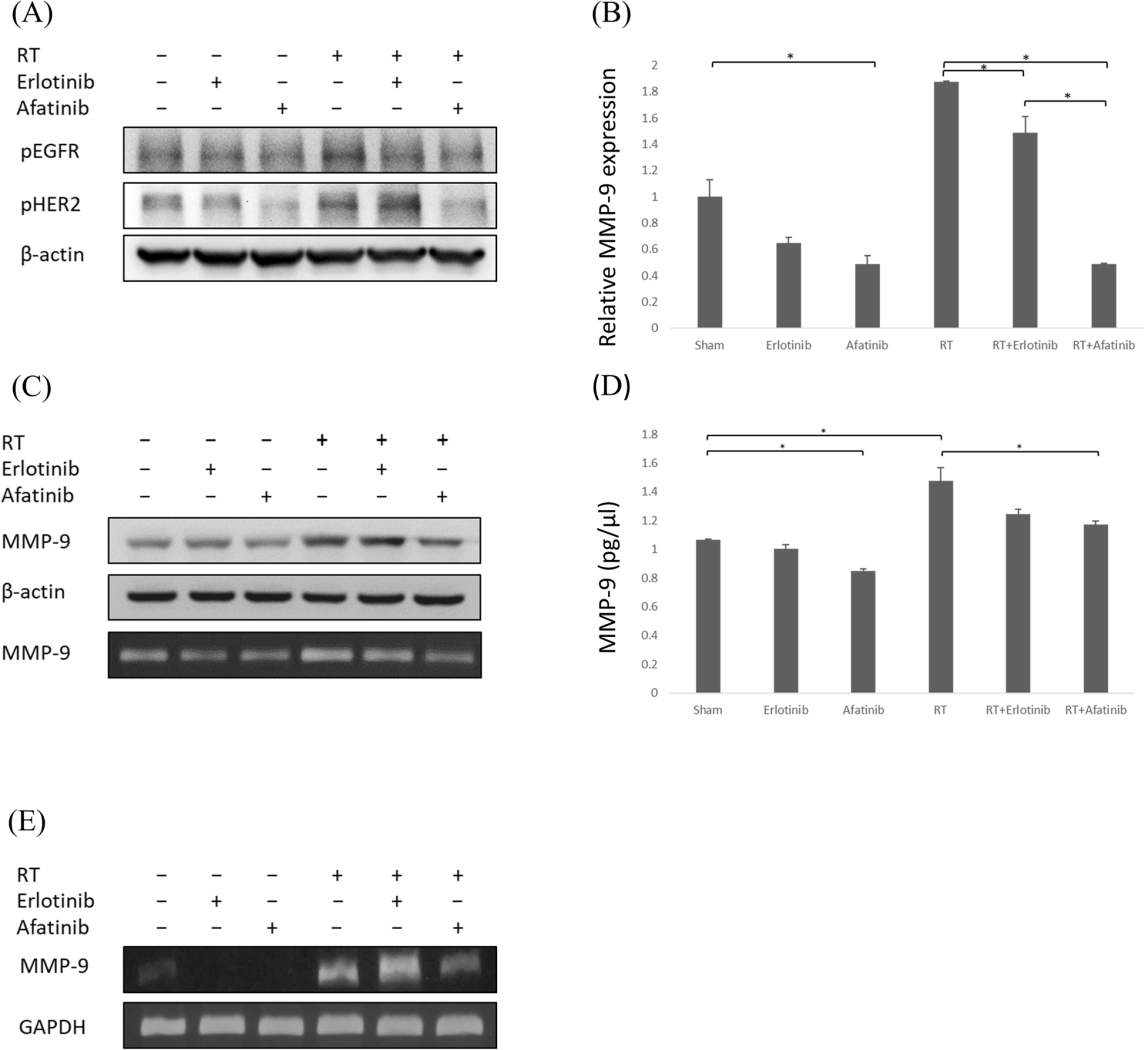
Inhibition of EGFR and HER2 tyrosine kinases suppresses radiation-activated MMP-9 expression. **a** LLC cells exposed or unexposed to radiation (RT) (7.5Gy) were treated with afatinib (100 nM), erlotinib (1 μM), or control. After 2 h, pEGFR and pHER2 in total cell lysates was detected by Western blotting, with β-actin as a loading control. RT increased both pEGFR and pHER2, which were inhibited by afatinib. But erlotinib only inhibited pEGFR. **b** RT-PCR assay showed that RT increased MMP-9 expression, which was significantly reduced by afatinib and erlotinib. Gene expression was measured relative to the sham control. * indicating *p* < 0.05. **c** After 12 h post-RT, MMP-9 in the total cellular lysate was detected by Western blotting; **d** The total MMP-9 concentrations in the culture supernatant were detected. * indicating *p* < 0.05; **e** MMP-9 activities were determined using gelatin zymography

Radiation increased the phosphorylation of both EGFR and HER2. Erlotinib reduced EGFR phosphorylation while afatinib reduced both EFGR and HER2 phosphorylation (Fig. 1a). In addition, radiation increased the amount of MMP-9 mRNA transcript (Fig. 1b), as well as protein expression (Fig. 1c), concentration (Fig. 1d), and activity (Fig. 1e). Compared to erlotinib, afatinib more effectively reduced the radiation-induced MMP-9 mRNA (*P* = 0.005), protein expression, and activity. These results indicated that the dual inhibition of EGFR and HER2 decreased MMP-9 transcription and translation in irradiated LLC cells.

### Dual blockade of EGFR and HER2 suppresses LLC cell invasiveness in vitro

**Fig. 2:**
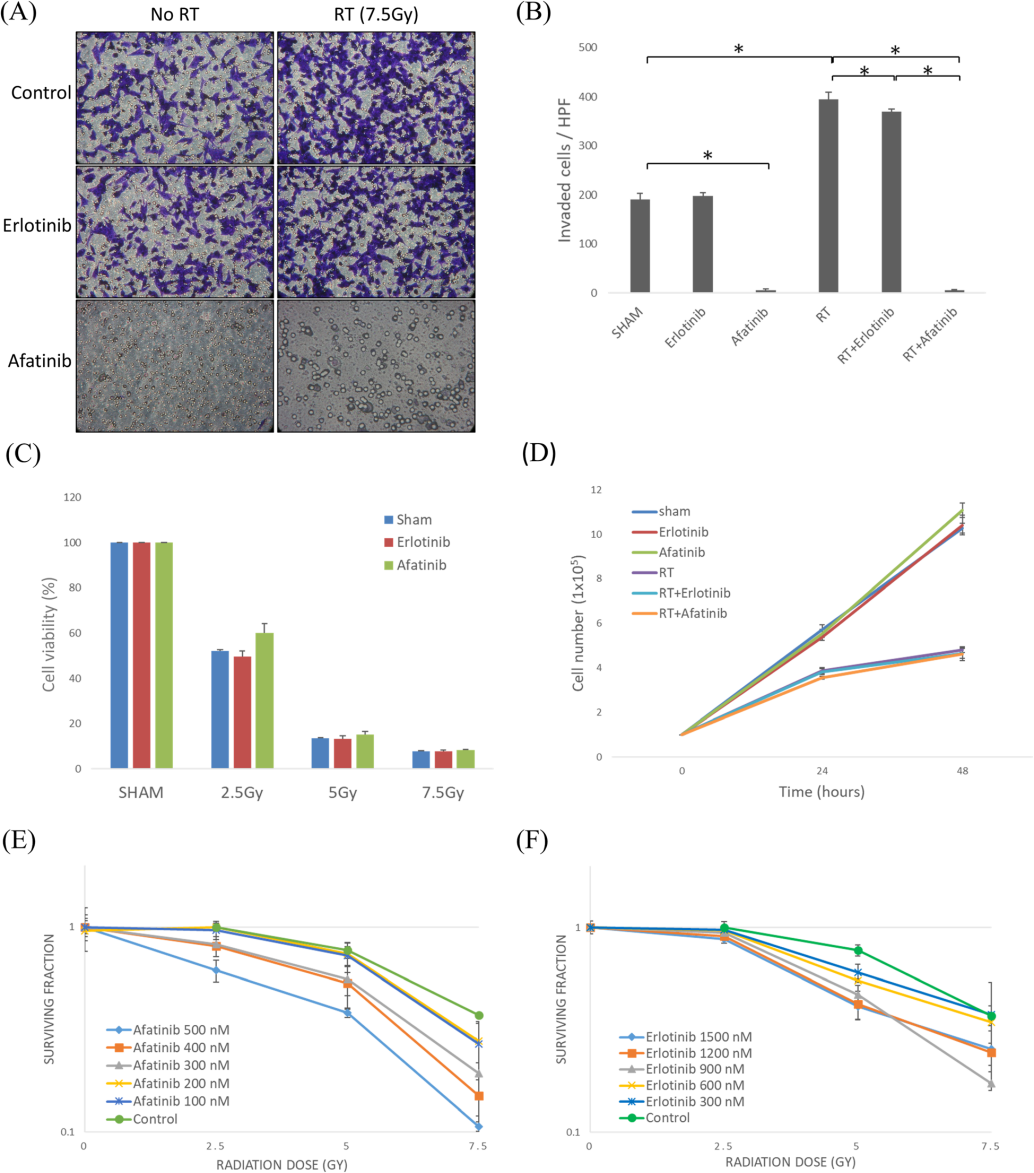
Dual blockade of EGFR and HER2 suppresses LLC cell invasiveness in vitro. **a** LLC cells were seeded in the Matrigel-coated inserts of Boyden chambers, and treated with sham radiation or radiation (RT) 7.5Gy and with erlotinib (1 μM), afatinib (100 nM), or control. After 24 h the invading cells were fixed, stained, and viewed by microscope (200X). **b** Invading cells were counted. * indicates *p* < 0.05. **c** LLC cells (10^5^ cells/dish) were seeded and irradiated with the indicated doses. The Trypan Blue assay was used to determine the percentage of viable cells at 24 h; **d** The number of viable cells was then determined 24 and 48 h later; **e** and **f** Quantitative results of clonogenic assays after combined treatment with either afatinib or erlotinib and RT (7.5Gy). The images (100X) were used to count colonies containing more than 50 cells. At each dose level, the colony count was expressed as a fraction of the number in the corresponding control group. Lines, mean (*n* = 3); Bars, S.D

Invasiveness of LLC cells in different treatment group were investigated through Boyden chamber invasion assay. LLC cell invasiveness was significantly enhanced after irradiation (Fig. 2a and b). Afatinib significantly reduced the invasion of both irradiated (*P* < 0.001) and non-irradiated cells (P < 0.001), whereas erlotinib was not effective as well. Radiation with or without afatinib showed no difference on cell viability at different radiation doses (Fig. 2c) and at 24 h and 48 h, respectively (Fig. 2d). The clonogenic assays of LLC cells after combined treatment with afatinib or erlotinib and radiation (0, 2.5, 5 and 7.5Gy) demonstrated that afatinib decreased the survival of LLC cells in a dose-dependent manner (Fig. 2e) while erlotinib had no effect (Fig. 2f). The results indicated that the dual inhibition of EGFR/HER2 with afatinib sensitizes LLC cells to radiation and reduces cell invasiveness.

### Genetic inhibition of HER2 reduced MMP-9 expression and LLC cell invasiveness

**Fig. 3:**
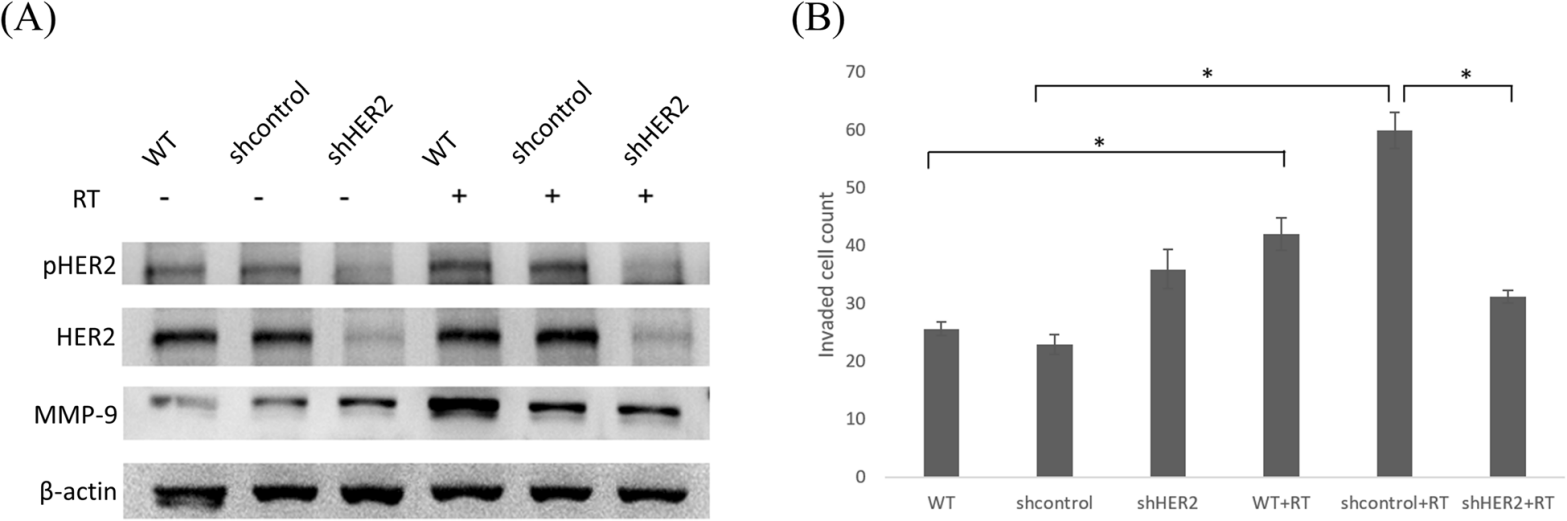
Knockdown of HER2 (shHER2) inhibits HER2 activation, MMP-9 expression, and LLC cell invasiveness. **a** The expression of pHER2, HER2, and MMP-9 in wild-type (WT), vector control (shcontrol), and shHER2 LLC cells were evaluated after irradiation (RT, 7.5Gy, 16 h) by Western blotting. **b** WT, shcontrol, and shHER2 LLC cells were seeded in the Matrigel-coated inserts of Boyden chambers, and treated with RT (7.5Gy) or not. After 24 h, the invaded cells were viewed and counted microscopically (40X). * indicates *p* < 0.05. Comparisons were made between the wild-type and wild-type+RT group; shcontrol and shcontrol+RT group; and shHER2 and shHER2 + RT group

To determine the effects of radiation-activated HER2 on MMP-9 expression and LLC cell invasiveness, cell lysates of HER2-KD LLC cells were prepared for Western blot analysis. HER2-KD reduced MMP-9 expression in radiated LLC cells and significantly reduced their invasiveness (*P* = 0.037) when compared to the irradiated vector-control cells (Fig. 3a and b). We subsequently compared the invasiveness of vector-control cells and HER2-knockdown cells pretreated with 30-μM zoledronic acid (Zobonic, TTY Biopharm Co., Ltd., Taipei, Taiwan), an MMP-9 inhibitory agent, and irradiated with 7.5 Gy. The effect on reducing invasiveness was better in vector-control than HER2-knockdown group (46.9% vs 78.6%) (Supplementary Figure 1).

### Afatinib inhibits radiation-induced Akt, ERK, and p38 phosphorylation in LLC cells

**Fig. 4:**
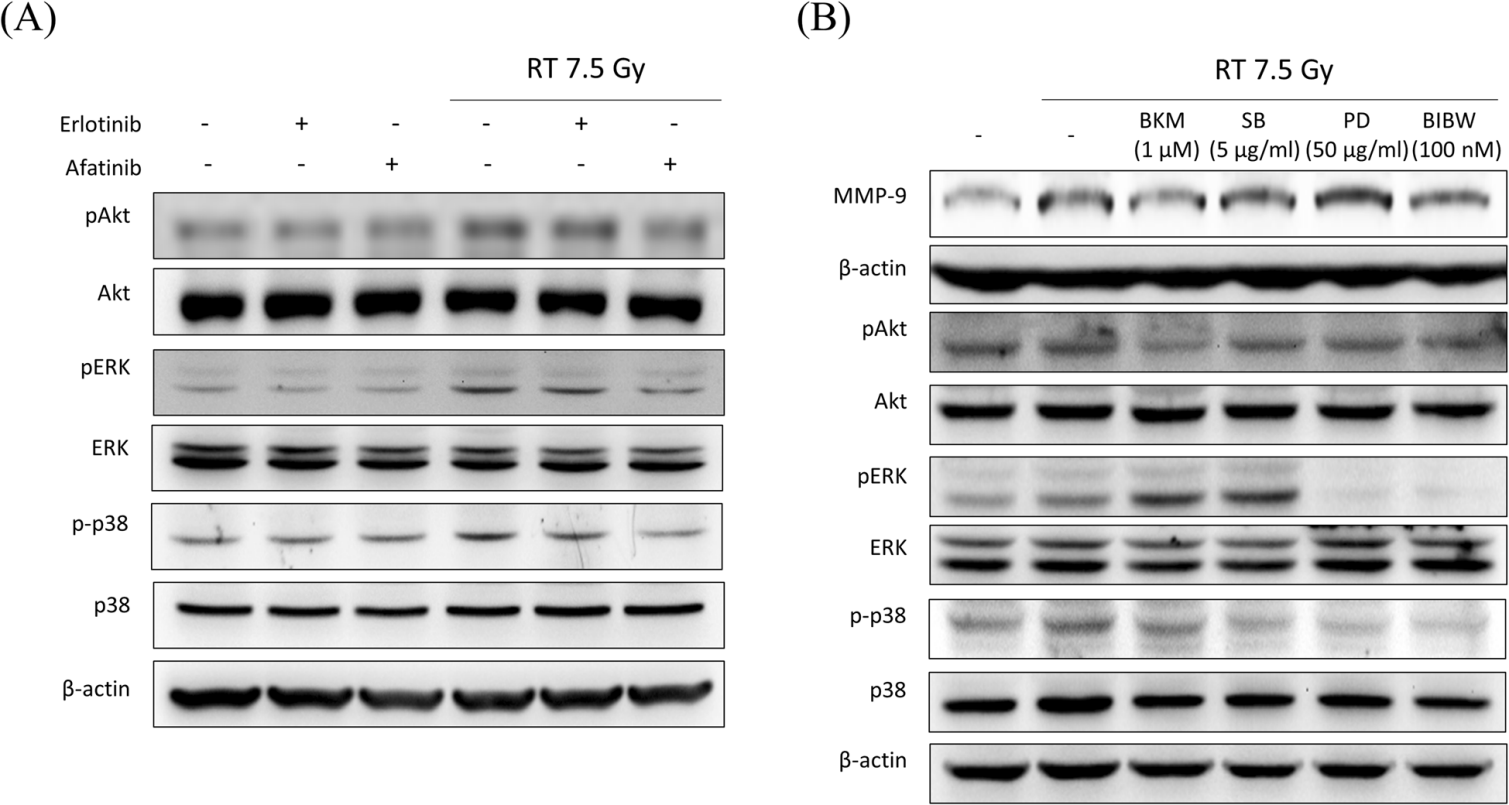
Afatinib inhibits radiation-induced Akt, ERK, and p38 phosphorylation in LLC cells. **a** Expression of Akt, ERK, and p38 in total cell lysates was detected by Western blotting. Radiation (RT) with 7.5 Gy increased the phosphorylation of Akt, ERK, and p38, which was better inhibited by afatinib than by erlotinib. **b** Expression of MMP-9, pAkt, pERK, and p-p38 in irradiated LLC cells pretreated with the PI3K inhibitor BKM120 (BKM, 1 μM), the p38 inhibitor SB203580 (SB, 5 μg/ml), the ERK inhibitor PD98059 (PD, 50 μg/ml), and afatinib (BIBW, 100 nM) were detected by Western blotting. Afatinib inhibited the phosphorylation of Akt, ERK, and p38, and the expression of MMP-9 as effectively as BKM and better than SB and PD

As downstream protein targets of EGFR and HER2 dimerization and activation, radiation-activated phosphorylation of Akt, ERK, and p38 was inhibited by afatinib but not by erlotinib (Fig. 4a).

### The inhibitory effects of afatinib on MMP-9 and EGFR downstream signaling are similar to those of PI3K inhibitors and more potent than those of p38 and ERK inhibitors

Lysates of LLC cells treated with indicated agents were prepared for the Western blot analysis of p-Akt, Akt, p-ERK, ERK, p-p38, p38, MMP-9 and β-actin. Afatinib effectively inhibited the radiation-activated phosphorylation of Akt, ERK, and p38 (Fig. 4b). Radiation-induced MMP-9 expression was reduced by afatinib and BKM120, but not by SB203580 or PD98059.

### Afatinib delays primary tumor growth and reduces radiation-enhanced LLC lung metastasis in vivo

**Fig. 5:**
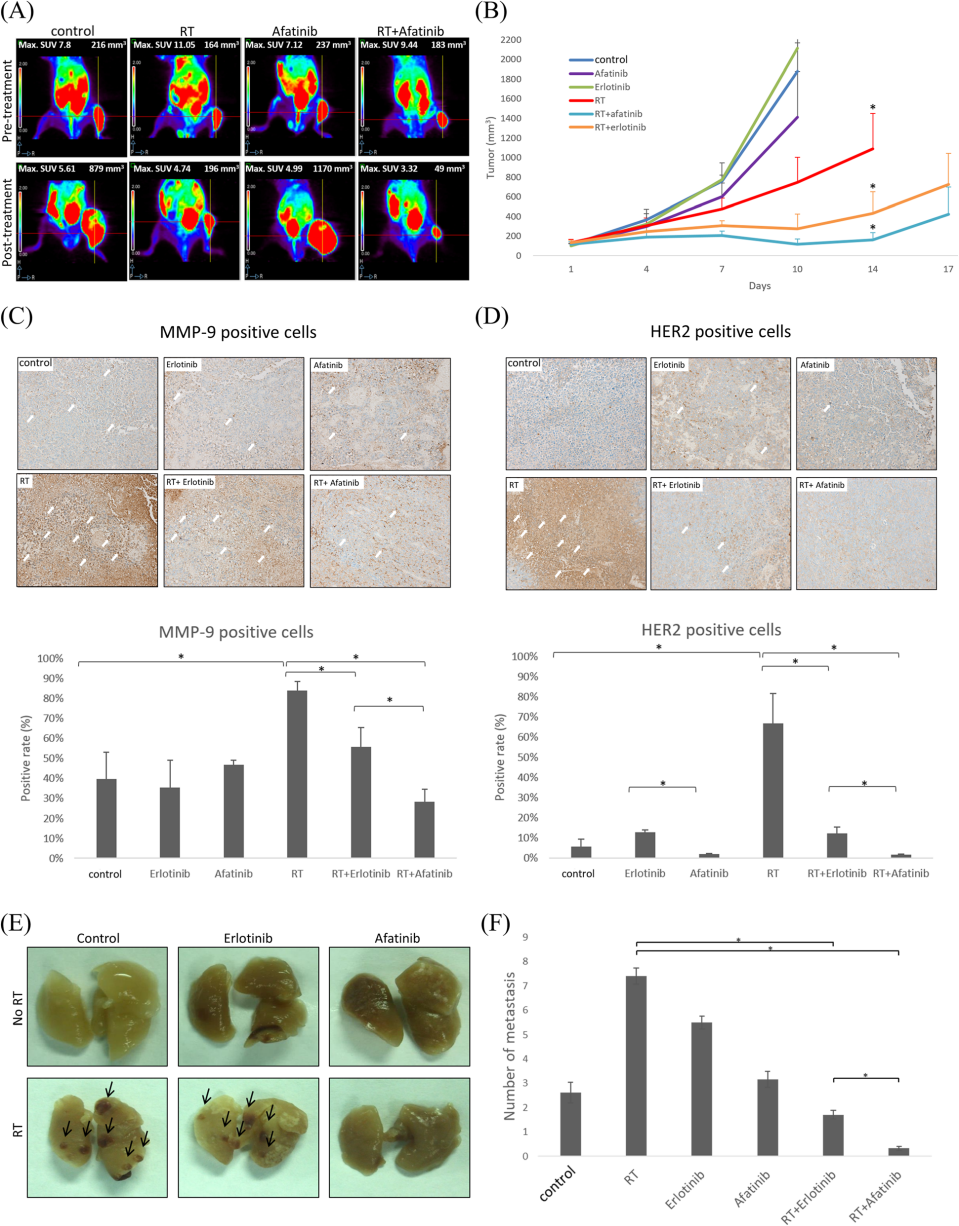
Afatinib delays ectopic tumor growth, reduces MMP-9, HER2 expressions, and radiation-enhanced lung metastases in mice. The mice were randomized into sham, radiotherapy (RT) with five 10-Gy treatments on days 8–12, afatinib, and afatinib+RT groups. **a** Primary tumor viability of one representative mouse from each treatment group was determined on day 7 (pre-RT) and day 14 (post-RT) by PET/CT. Representative images are shown. Crosshairs indicate the viable right thigh tumors. The maximum standard uptake value and the viable tumor volume are shown at the left and right corners of the image, respectively. **b** The tumor growth curves in the different treatment groups were plotted. The data points are the mean tumor volume from each group measured on the indicated days. *P* < 0.05 was considered statistically significant for the cross comparison of RT, RT + afatinib and RT + erlotinib groups on day 14. Microscopic images (200X) are shown of immunohistochemically stained tumor tissue sections with **c** MMP-9 and **d** HER2 from the different treatment groups with white arrows indicating the positively stained cells. The percentage of MMP-9- and HER2-positive cells was calculated by dividing the number of positive cells by the total number of cells in the representative fields. * indicates *p* < 0.05. **e** Representative sets of lungs with surface metastases (arrow) from each treatment group. **f** The numbers of surface metastases to the lungs were counted. * indicates *p* < 0.05, RT vs RT + erlotinib group, RT vs RT + afatinib group, RT + erlotinib vs RT + afatinib group

In Fig. 5a, the threshold-uptake volume and the standard uptake value of the primary tumor grafted on murine thigh were much smaller with combined afatinib and RT. While the combination of erlotinib and RT significantly suppressed tumor growth, combination of afatinib and RT more effectively suppressed tumor growth than either RT or RT with erlotinib. (Fig. 5b) The mean tumor volumes on the fourteenth day were 1087 ± 363 mm^3^ and 162 ± 74 mm^3^ in the mice receiving RT and combined afatinib+RT, respectively (*P* = 0.003). HER2 (Fig. 5c) and MMP-9 (Fig. 5d) expressions in wild-type tumors with different treatments were evaluated using immunohistochemical staining of xenograft tissues. MMP-9 and HER2 expressions were significantly induced by RT (*P* < 0.0001 and *P* < 0.0001, respectively). Both MMP-9 and HER2 expressions were significantly reduced by combined afatinib and RT (*P* < 0.0001 and P < 0.0001, respectively), with the combined erlotinib less effective (*P* < 0.0005 and *P* < 0.0005, respectively). Moreover, the number of pulmonary metastases was significantly lower with combined afatinib and RT, as compared to RT alone (*P* = 0.008) or combined erlotinib and RT (*P* = 0.045) (Fig. 5e and f).

### HER2 knock-down sensitizes LLC tumors to radiotherapy and reduces tumor growth rate

**Fig. 6:**
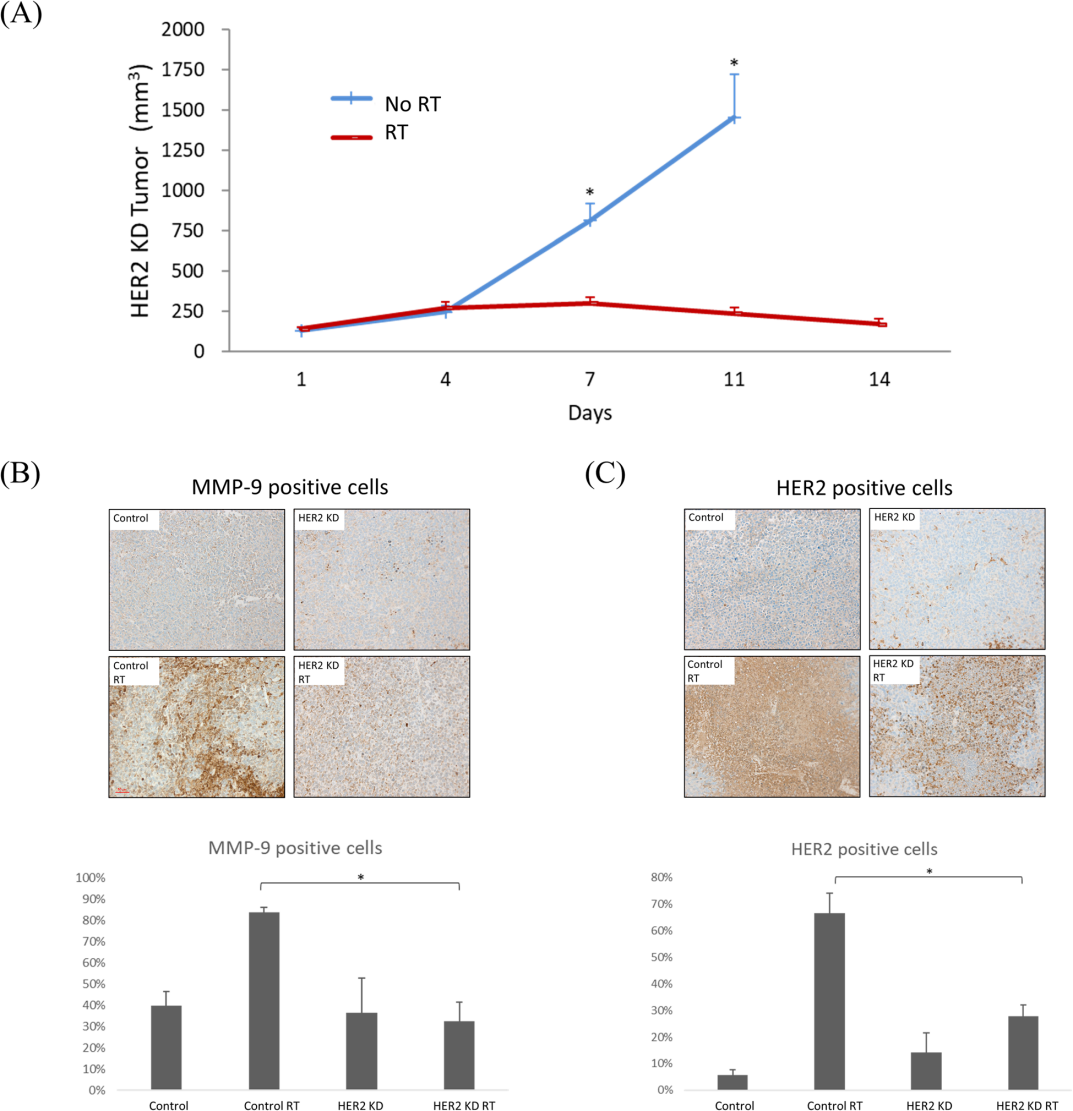
Knockdown of HER2 (HER2-KD) significantly sensitizes LLC cells to radiotherapy (RT) in a mouse model. **a** HER2-KD LLC cells (1 × 10^6^) were injected subcutaneously into the right hind limb of B57CL/6 mice and received five 10-Gy radiotherapy (RT) doses on days 4–8. Microscopic images (200X) are shown of immunohistochemically stained tumor tissue sections with **c** MMP-9 and **d** HER2 from the different treatment groups. * indicates *p* < 0.05, wild-type RT vs HER2-KD RT group

RT significantly delayed the growth of HER2-KD LLC tumors compared to the un-irradiated tumors (Fig. 6a). The mean tumor volume on the eleventh day was 1457 ± 668 mm^3^ and 236 ± 97 mm^3^ in the HER2-KD tumors treated without and with RT, respectively. The size differences were all statistically significant on day 7 (*P* = 0.002) and day 11 (*P* = 0.003). Furthermore, both MMP-9 (Fig. 6b) and HER2 (Fig. 6c) expressions in the HER2-KD xenograft tissues were significantly reduced in irradiated HER2-KD tumors compared with irradiated wild-type tumors (*P* < 0.0001 and *P* < 0.0001, respectively).

## Discussion

NSCLC is one of the leading causes of cancer-related mortality worldwide. More than 60% of the patients have unresectable disease when they are diagnosed. In NSCLC, the overexpression of the EGFR and HER2 proto-oncogenes is closely associated with tumor progression, treatment resistance, invasion, and metastasis [4, 15]. In published meta-analyses on NSCLC patients, HER2 overexpression was associated with poor prognosis, and a prognostic impact was confirmed in up to 35% of the patients [16–18]. The standard treatment for unresectable locally advanced NSCLC is RT combined with concomitant chemotherapy [19, 20]. The current treatment paradigm is ineffective, as the majority of the patients succumb to distant metastatic dissemination. Previous study showed that MMP-9 derived from sublethally irradiated lung carcinoma cells plays an important role in radioresistance and in initiating metastatic cascades [12]. Furthermore, other studies have shown that not only EGFR expression up-regulates the MMP-9 production, radiation stimulated HER2 and EGFR heterodimerization also activates the AKT signaling pathway and eventually increases MMP-9 production [21]. In this study, we underscore the crucial role of HER2 expression in MMP-9-mediated radiation-induced LLC cell invasiveness and metastasis. Though Afatinib or Erlotinib alone showed no significant difference in tumor cells proliferation activity. Compared to the inhibition of EGFR by erlotinib, the dual inhibition of EGFR/HER2 by afatinib more effectively suppressed MMP-9 transcription and translation in vitro. HER2 inhibition by afatinib or the genetic knockdown of HER2 effectively mitigated cell invasiveness, radioresistance, and metastases of the irradiated LLC tumors in vivo.

The EGFR/HER2 signaling network is pivotal in controlling cancer proliferation and metastasis through the downstream effectors of AKT, ERK, and STAT3 [22–24]. Furthermore, hyperactivated HER2 signaling upregulates the cyclin D complex, promoting tumor cell invasion and metastasis [25, 26]. Afatinib decreases phosphorylation between ErbB dimers more effectively than erlotinib and it has been reported to overcome therapy resistance to EGFR TKI in lung cancer clinically [27, 28]. The off-target effect of afatinib on tumor metastasis cannot be underestimated and has been reported in previous studies [29]. Besides the inhibition of MMP-9, MMP-2 expression and the ratio of Bax/Bcl-2 decreased evidently with increasing afatinib concentrations. MMP-2 was reported to be a determinant of metastatic potential for cancer cell. The decreased Bax expression was associated with distant metastases and a more infiltrative growth pattern in colorectal cancer [30]. Furthermore, in patients with advanced NSCLC harboring common EGFR mutation (Del19/L858R), the overall survival improved with first-line afatinib use over chemotherapy [31]. Previous studies have reported variable radiosensitizing effects of afatinib in different cell lines, including NSCLC cells with gefitinib resistant mutation, hypopharyngeal carcinoma cells and glioma cells [32–34]. Although EGFR TKIs have been reported with the inhibitory activity on HER2 and MMP-9 at much higher concentrations [35, 36], our data showed a less potent radiosensitizing effect of 1-μM erlotinib than 100-nM afatinib.

The LLC cell line is primarily used to model metastasis and evaluate the efficacy of therapeutic agents in vivo [37]. Although the cell death following mitotic catastrophe induced by irradiation may occur up to 6 days following irradiation, invasiveness and tumor metastasis may develop within 48 h after irradiation [38]. Thus, the early inhibition of cancer cell metastasis in the first 48 h post-RT is critical for improving therapeutic outcomes. In our study, afatinib, but not erlotinib, reduced MMP-9 expression in cell lysates 12 h following irradiation. The result implies that afatinib is able to reduce the metastatic potential of LLC cells in the early phases after RT. This finding emphasizes the importance of pretreatment with afatinib before RT, especially in a cell line overexpressing HER-2, to prevent initiating the metastatic cascade before the death of the primary irradiated tumor.

This study has a few limitations. First, afatinib may exert off-target effects on protein kinases other than EGFR and HER2. The potential off-target effects of afatinib may need to be tested in HER2-KD LLC cells. Second, angiogenesis has been one of the key mechanisms that mediate radiation-activated pulmonary metastasis. The association between HER2 signaling and angiogenesis cascade warrants further elucidation. Lastly, our findings in murine tumor model may not be fully translated to human lung cancer.

## Conclusions

Afatinib is more effective than erlotinib in reducing survival and invasiveness of irradiated LLC cells in vitro by inhibiting cell proliferation/viability, deactivating the EGFR/HER2 signaling proteins, and partly by decreasing MMP-9 production. In mice, the pharmacological or genetic HER2 inhibition enhanced tumor control and decreased the metastatic potential of the irradiated LLC tumors. Therefore, targeting HER2 can effectively improve the response to RT in lung cancer and prevent subsequent metastatic cascades.

## Supplementary information

**Additional file 1: Figure S1.** LLC cells divided into HER2-knockdown group and vector-control group were seeded in the Matrigel-coated inserts of Boyden chambers, and treated with sham radiation (no RT) or radiation 7.5Gy (RT) and with or without zoledronic acid (ZA, 30 μM). After 24 h the invading cells were fixed, stained, and viewed by microscope (200X). Invading cells were counted. The effect of MMP9 inhibition on reducing invasiveness were presented by the ratio of invaded cell counts in groups with ZA to groups without ZA.

## Data Availability

The datasets used and/or analysed during the current study are available from the corresponding author on reasonable request.

## Abbreviations

CT: Computed tomography
EGFR: Epidermal growth factor receptor
FDG: [18F]-2-fluoro-2-deoxyD-glucose
HER2-KD: HER2 knockdown
LLC: Lewis lung carcinoma
MMP-9: Matrix metalloproteinase 9
PET: Positron emission tomography
RT: Radiation therapy
TKIs: Tyrosine kinase inhibitors
