# Supplementary figures and images for: Targeting human epidermal growth factor receptor 2 enhances radiosensitivity and reduces the metastatic potential of Lewis lung carcinoma cells
Source: Radiat Oncol. 2020 Mar 6;15:58. doi: 10.1186/s13014-020-01493-8 (PMC7060577; doi:10.1186/s13014-020-01493-8)

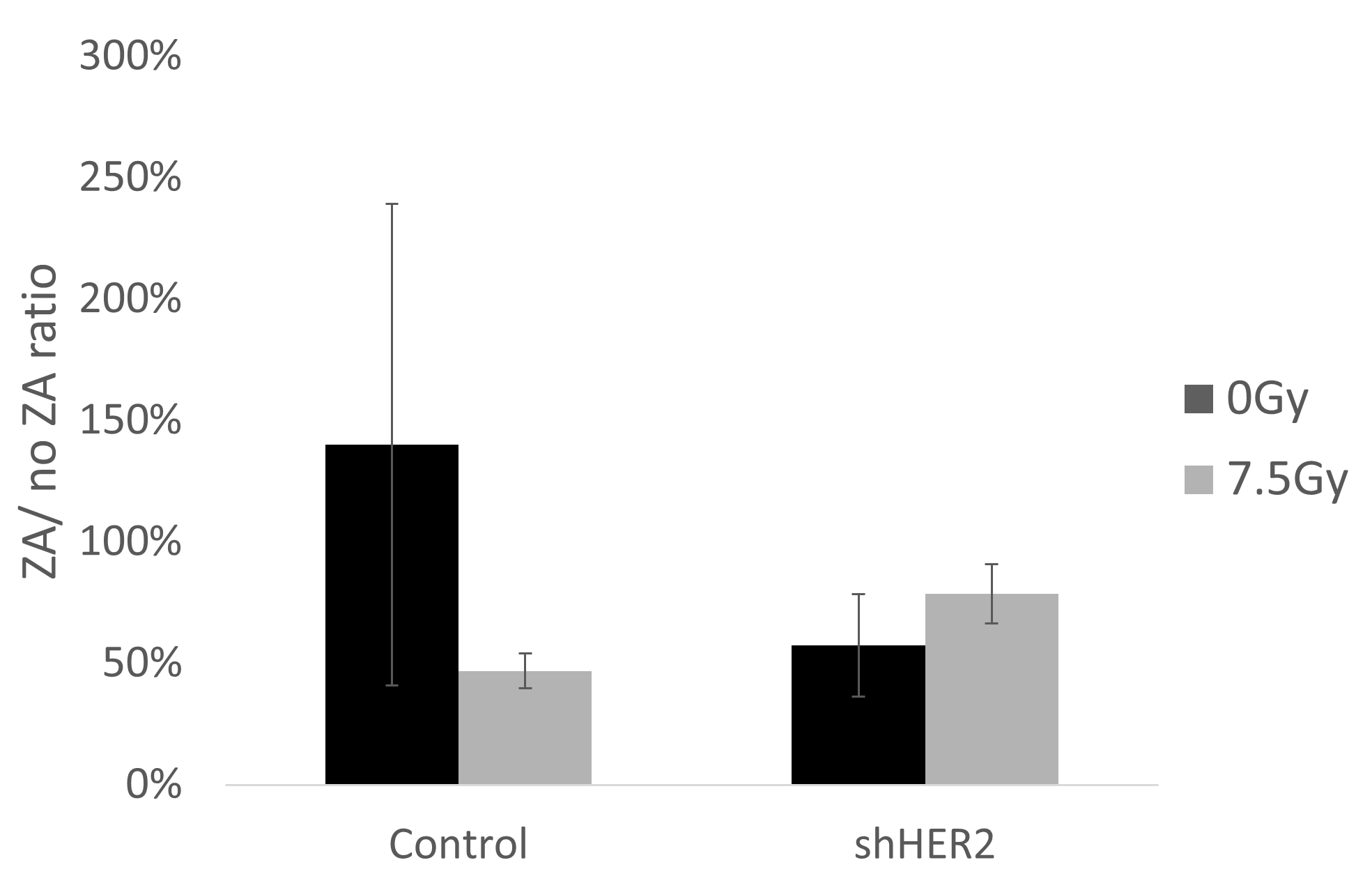

Supplement: Supplementary file 1 — Additional file 1: Figure S1. LLC cells divided into HER2-knockdown group and vector-control group were seeded in the Matrigel-coated inserts of Boyden chambers, and treated with sham radiation (no RT) or radiation 7.5Gy (RT) and with or without zoledronic acid (ZA, 30 μM). After 24 h the invading cells were fixed, stained, and viewed by microscope (200X). Invading cells were counted. The effect of MMP9 inhibition on reducing invasiveness were presented by the ratio of invaded cell counts in groups with ZA to groups without ZA. [file 13014_2020_1493_MOESM1_ESM.zip › Supplementary Figure 1.tif]
